# A Unique Role for the Host ESCRT Proteins in Replication of *Tomato bushy stunt virus*

**DOI:** 10.1371/journal.ppat.1000705

**Published:** 2009-12-24

**Authors:** Daniel Barajas, Yi Jiang, Peter D. Nagy

**Affiliations:** Department of Plant Pathology, University of Kentucky, Lexington, Kentucky, United States of America; Oregon Health and Science University, United States of America

## Abstract

Plus-stranded RNA viruses replicate in infected cells by assembling viral replicase complexes consisting of viral- and host-coded proteins. Previous genome-wide screens with *Tomato bushy stunt* tombusvirus (TBSV) in a yeast model host revealed the involvement of seven ESCRT (endosomal sorting complexes required for transport) proteins in viral replication. In this paper, we show that the expression of dominant negative Vps23p, Vps24p, Snf7p, and Vps4p ESCRT factors inhibited virus replication in the plant host, suggesting that tombusviruses co-opt selected ESCRT proteins for the assembly of the viral replicase complex. We also show that TBSV p33 replication protein interacts with Vps23p ESCRT-I and Bro1p accessory ESCRT factors. The interaction with p33 leads to the recruitment of Vps23p to the peroxisomes, the sites of TBSV replication. The viral replicase showed reduced activity and the minus-stranded viral RNA in the replicase became more accessible to ribonuclease when derived from *vps23Δ* or *vps24Δ* yeast, suggesting that the protection of the viral RNA is compromised within the replicase complex assembled in the absence of ESCRT proteins. The recruitment of ESCRT proteins is needed for the precise assembly of the replicase complex, which might help the virus evade recognition by the host defense surveillance system and/or prevent viral RNA destruction by the gene silencing machinery.

## Author Summary

Plus-stranded RNA viruses, which are important pathogens of humans, animals and plants, replicate in infected cells by assembling viral replicase complexes consisting of viral- and host-coded proteins. In this paper, we show that a group of host factors called ESCRT proteins (endosomal sorting complexes required for transport) play important roles in tombusvirus replication. The expression of dominant negative mutants of ESCRT factors inhibited virus replication in the plant host, suggesting that tombusviruses co-opt selected ESCRT proteins for the assembly of the viral replicase complex. In addition, we show direct interaction between the viral p33 replication protein and Vps23p ESCRT-I and Bro1p accessory ESCRT factors. The interaction with p33 leads to the recruitment of Vps23p to the peroxisomes, the sites of tombusvirus replication. We also showed that the viral RNA within the viral replicase complex became more sensitive to ribonuclease in the absence of ESCRT factors, suggesting that the protection of the viral RNA is compromised within the replicase complex assembled in the absence of ESCRT proteins. Intriguingly, the host ESCRT factors also affect the budding of several enveloped viruses, intracellular transport of proteins and cytokinesis. Overall, this work demonstrates that a plus-stranded RNA virus uses the endosomal sorting pathway in a unique way.

## Introduction

Plus-stranded (+)RNA viruses replicate in the infected cells by assembling viral replicase complexes consisting of viral- and host-coded proteins in combination with the viral RNA template. Although major progress has recently been made in understanding the functions of the viral replication proteins, including the viral RNA-dependent RNA polymerase (RdRp) and auxiliary replication proteins, the contribution of host proteins is poorly documented [1],[2],[3],[4]. Genome-wide screens to identify host factors affecting (+)RNA virus infections, such as *Brome mosaic virus* (BMV), *Tomato bushy stunt virus* (TBSV), West Nile virus and Droshophila virus C, in yeast and animal model hosts led to the identification of host proteins including ribosomal proteins, translation factors, RNA-modifying enzymes, proteins of lipid biosynthesis and others [2],[3],[5],[6],[7],[8],[9]. The functions of the majority of the identified host proteins in (+)RNA virus replication have not been fully revealed.

TBSV is a small (+)RNA virus that infects a wide range of host plants. TBSV has recently emerged as a model virus to study virus replication, recombination, and virus - host interactions due to the development of yeast (*Saccharomyces cerevisiae*) as a model host [10],[11],[12],[13]. Systematic genome-wide screens covering 95% of yeast genes have led to the identification of over 100 host genes that affected either TBSV replication or recombination [5],[7],[14],[15]. Moreover, proteomics analysis of the highly purified tombusvirus replicase complex revealed the presence of the two viral replication proteins (i.e., p33 and p92^pol^) and 6–10 host proteins in the replicase complex [16],[17],[18]. These host proteins have been shown to bind to the viral RNA and the viral replication proteins [1],[17],[19]. The auxiliary p33 replication protein has been shown to recruit the TBSV (+)RNA to the site of replication, which is the cytosolic surface of peroxisomal membranes [20],[21],[22]. The RdRp protein p92^pol^ binds to the essential p33 replication protein that is required for assembling the functional replicase complex [12],[22],[23],[24].

Genome-wide screens for host factors affecting TBSV replication in yeast [5],[7] has led to the identification of seven ESCRT proteins involved in multivesicular body (MVB)/endosome pathway [25],[26]. The identified host proteins included Vps23p and Vps28p (ESCRT-I complex), Snf7p and Vps24p (ESCRT-III complex); Doa4p ubiquitin isopeptidase, Did2p having Doa4p-related function; and Vps4p AAA-type ATPase [5]. The identification of ESCRT proteins supports the idea that tombusvirus replication could depend on hijacking of ESCRT proteins, thus promoting efforts to test their roles in TBSV replication in this paper. Recruitment of ESCRT proteins for TBSV replication might facilitate the assembly of the replicase complex, including the formation of TBSV-induced spherules and vesicles in infected cells [27]. Induction of membranous spherule-like replication structures in infected cells might be common for many plus-stranded RNA viruses [28].

The endosome pathway is a major protein-sorting pathway in eukaryotic cells, which down- regulates plasma membrane proteins via endocytosis; and sorts newly synthesized membrane proteins from trans-Golgi vesicles to the endosome, lysosome or the plasma membrane [29],[30],[31]. The ESCRT proteins in particular have a major role in sorting of cargo proteins from the endosomal limiting membrane to the lumen via membrane invagination and vesicle formation. Defects in the MVB pathway can cause serious diseases, including cancer, defect in growth control and early embryonic lethality [29],[30],[31],[32]. In addition, various viruses, such as enveloped retroviruses (HIV), (+) and (−)RNA viruses (such as filo-, arena-, rhabdo and paramyxoviruses) usurp the MVB pathway by redirecting ESCRT proteins to the plasma membrane, leading to budding and fission of the viral particles from infected cells [25],[26].

The first step in the endosome pathway is the monoubiquitination of cargo proteins, which serves as a signal for proteins to be sorted into membrane microdomains of late endosomes [30],[31],[32]. The ubiquitinated cargo protein is bound by Vps27p (Hrs protein in mammals), which in turn recruits Vps23-containing ESCRT-I complex. Then, the ESCRT-I-complex recruits ESCRT-II complex, which in turn recruits the large ESCRT-III complex. The proposed role of the ESCRT-III complex is grouping the cargo proteins together in the limiting membranes of late endosomes and deforming the membranes that leads to membrane invagination into the lumen [33],[34]. Then, Vps4p recycles the ESCRT proteins, whereas Doa4p recycles the ubiquitin, leading to budding of multiple small vesicles into the lumen of endosome and to MVB formation. Fusion of the limiting membrane of MVB with the lysosome/vacuole will release the lipid/protein content of MVB into the lysosome, or alternatively, by fusing to the plasma membrane (exocytosis), it releases its content outside the cell [30],[31],[32].

In this paper, we show that ESCRT proteins previously identified in a genome-wide screen in yeast for affecting TBSV replication are involved in tombusvirus replication in plants. We demonstrate that Vps23p, which is a key ESCRT-I adaptor protein recognizing ubiquitinated cargo proteins, and Bro1p accessory ESCRT protein bind to the TBSV p33 replication protein, which could be critical for p33 replication protein to recruit Vps23p and Bro1p, allowing TBSV to usurp additional ESCRT proteins for replication. We also show that functional ESCRT proteins are needed for optimal replicase activity and protection of the viral RNA template within the tombusvirus replicase from a ribonuclease *in vitro*. These data are consistent with the model that TBSV co-opts ESCRT proteins for its replication.

## Results

### Inhibition of tombusvirus replication in *N. benthamiana* by expression of dominant negative mutants of ESCRT factors

To test the roles of ESCRT proteins in tombusvirus replication in a plant host, we have expressed dominant negative mutants of selected ESCRT factors in *N. benthamiana*. This approach has been facilitated by the availability of dominant negative mutants of *ESCRT* genes in mammals and yeast [25],[35]. Expression of dominant negative mutants might inhibit the function of the endogenous ESCRT proteins in *N. benthamiana*, albeit the number of *VPS23* and other *ESCRT* genes are not known in *N. benthamiana*, whose genome is not yet sequenced completely.

**Figure 1:**
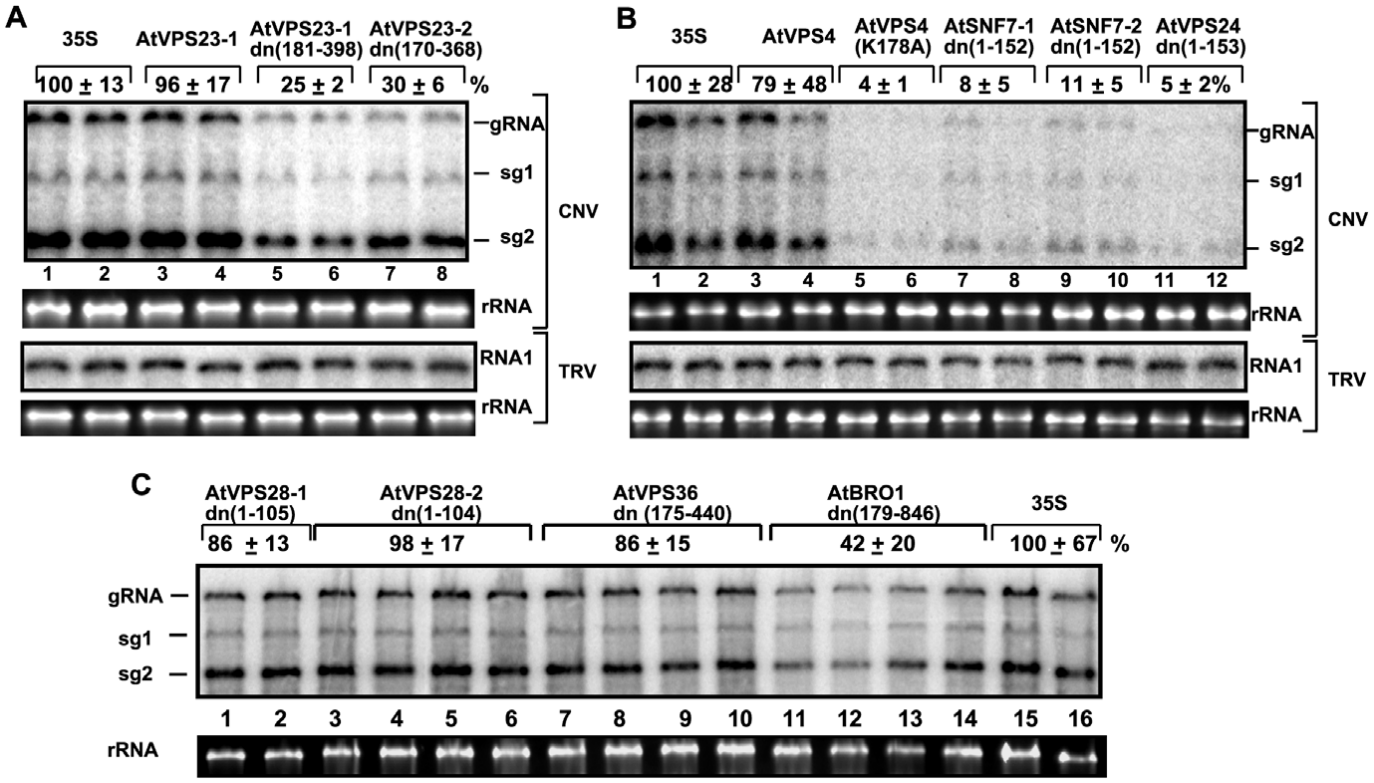
Inhibition of tombusvirus RNA accumulation in plants by expression of dominant negative ESCRT mutants. (A) Expression of N-terminal deletion mutants of the two homologous AtVps23p proteins (lanes 5–8) was done in *N. benthamiana* leaves, which were co-infiltrated with *Agrobacterium* carrying a plasmid to launch CNV replication from the 35S promoter. The control samples were obtained from leaves expressing no proteins (35S, lanes 1-2) or the full-length AtVps23p (lanes 3–4). Total RNA was extracted from leaves 2.5 days after agroinfiltration. The accumulation of CNV gRNA and subgenomic (sg)RNAs in *N. benthamiana* leaves was measured by Northern blotting (Top panel). The ribosomal RNA (rRNA) was used as a loading control and shown in agarose gel stained with ethidium-bromide (Second panel). The bottom two panels show the lack of inhibition of TRV RNA1 accumulation by the expression of the above proteins. TRV infection was launched by agroinfiltration as described above for CNV. (B) Blocking tombusvirus RNA replication in plants by expression of the dominant negative mutant of AtVps4p [named AtVps4p(K_178_A)] (lanes 5–6), and C-terminal deletion mutants of two homologous AtSnf7p and one AtVps24p ESCRT-III proteins. See further details in panel A. (C) The expression of the C-terminal deletion mutants of two homologous AtVps28p and the N-terminal deletion mutants of AtVps36p and AtBro1p ESCRT proteins in plants was done from the constitutive 35S promoter. See further details in panel A.

To this end, we cloned ten ESCRT genes, including *AtVPS4, AtVPS24, AtVPS36, AtBRO1,* and two genes for *AtVPS23, AtVPS28,* and *AtSNF7* from *Arabidopsis thaliana*, a model plant with known sequence, which is not infected by TBSV or the closely related *Cucumber necrosis virus* (CNV). First, we have made dominant negative mutants of two *AtVPS23* via deletion of the N-terminal UEV (ubiquitin E2 variant) domain. We have found that co-expression of CNV genomic (g)RNA with either *AtVPS23-1*dn or *AtVPS23-2*dn in *N. benthamiana* leaves from the constitutive 35S promoter via agroinfiltration led to inhibition of CNV gRNA replication in the infiltrated leaves (down to 25–30%, Fig. 1A, lanes 5–8). Expression of the wt *AtVPS23-1* in *N. benthamiana* did not inhibit CNV RNA accumulation when compared with the samples based on agroinfiltration with the empty vector (Fig. 1A, lanes 3-4 versus 1-2). Altogether, inhibition of CNV gRNA replication by the dominant negative Vps23p mutants supports the idea that Vps23p play a significant role in tombusvirus replication in a plant host.

To test the effect of additional dominant negative mutants in the ESCRT pathway, we generated a dominant negative mutant of *AtVPS4* by changing the highly conserved K178 to A, which has been shown to inhibit the ATPase activity required for disassembly and release of the ESCRT proteins from the endosomal membranes, resulting in strong inhibition of ESCRT functions [25],[35]. We have found that co-expression of CNV with *AtVPS4(K178A)* in *N. benthamiana* leaves led to dramatic inhibition of CNV gRNA replication in the infiltrated leaves (down to 4%, Fig. 1B, lanes 5–6), whereas expression of the wt *AtVPS4* in *N. benthamiana* inhibited CNV RNA accumulation only by 21% (Fig. 1B, lanes 3–4). In addition, expression of one *AtVPS24* gene and two *AtSNF7* genes with C-terminal deletions, which are known to interfere with proper ESCRT-III functions [36], inhibited CNV gRNA accumulation by 89–95% in the infiltrated leaves (Fig. 1B). Expression of *AtBRO1* mutant inhibited CNV replication to a lesser extent in *N. benthamiana* (by ∼60% Fig. 1C, lanes 11–14), whereas *AtVPS28* and *AtVPS36* mutants did not significantly affect CNV replication (Fig. 1C, lanes 1–10). Altogether, these data support the model that several ESCRT components are involved in tombusvirus replication in plants.

Since the ESCRT proteins are involved in membrane bending/invagination [33],[34], it is possible that they are used by tombusviruses during the assembly of the membrane-bound replicase complexes [1]. Accordingly, we observed the formation of the characteristic spherule-like structures (which likely serve as the sites of tombusvirus replication) in reduced number in plants actively replicating TBSV repRNA and expressing the dominant negative mutants *AtVPS4(K178A)* and *AtSNF7-1*, respectively, in *N. benthamiana* leaves when compared with the control samples (Fig. S1 and Protocol S1).

To exclude the possibility that the above inhibitory effect of the ESCRT protein mutants on tombusvirus replication is due to unwanted cytotoxic effect of the expressed ESCRT proteins, we tested the replication of a distantly related RNA virus, *Tobacco rattle virus* (TRV), in similarly treated *N. benthamiana* leaves. These experiments revealed the lack of inhibition of TRV RNA accumulation in leaves expressing the dominant negative ESCRT proteins (Fig. 1A-B, lower panels), suggesting that the inhibitory effects of these mutant proteins are specific to CNV RNA replication.

### Inhibition of *in vitro* tombusvirus replicase activity derived from plants expressing dominant negative mutants of ESCRT factors

**Figure 2:**
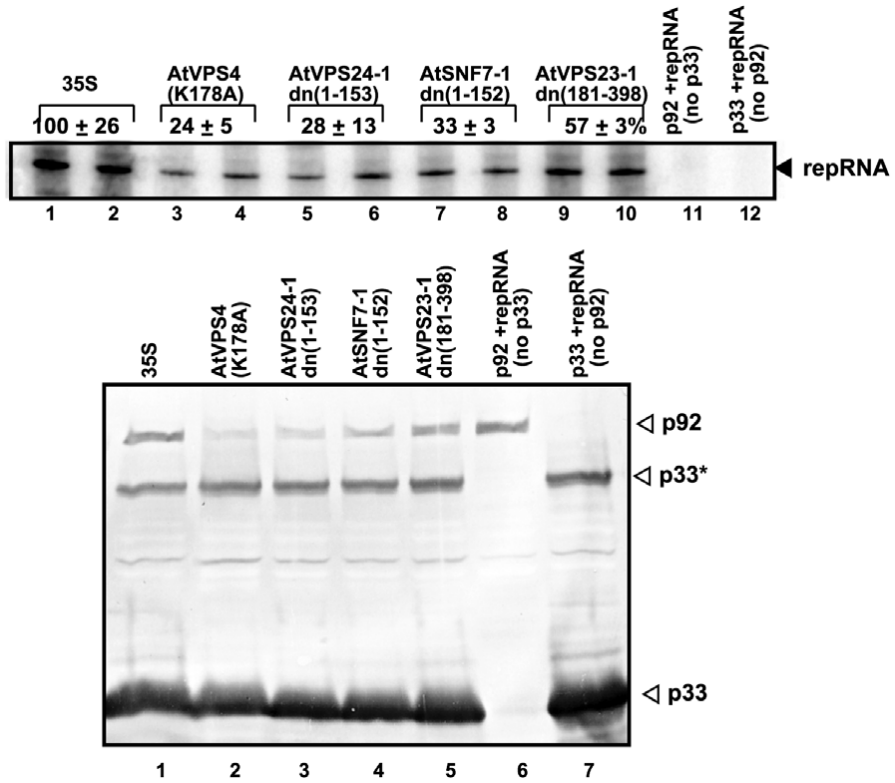
The isolated tombusvirus replicase preparations from *N. benthamiana* plants expressing dominant negative ESCRT factors show low activity *in vitro*. Top panel: Denaturing PAGE of *in vitro* replicase activity in the membrane-enriched fraction from co-infiltrated leaves expressing p33, p92^pol^, DI-72 repRNA, p19 (suppressor of gene silencing) and the shown dominant negative ESCRT factors using the co-purified repRNA template. The lack of replicase activity in the absence of p33 (lane 11) or p92^pol^ (lane 12) demonstrates that the *in vitro* replication is tombusvirus specific. Bottom panel: Western blot analysis of p33 and p92^pol^ levels in the above membrane-enriched fractions was performed with anti-p33 antibody.

To test if the above dominant negative ESCRT mutants affect the activity of the tombusvirus replicase, we isolated the membrane-bound tombusvirus replicase from *N. benthamiana* expressing selected mutated ESCRT proteins. The tombusvirus replication proteins as well as the plus-stranded (+) DI-72 replicon (rep)RNA were expressed from separate expression plasmids in the above plants. The activity of the isolated tombusvirus replicase was tested *in vitro* on the co-purified repRNA (Fig. 2). These experiments revealed that the expression of dominant negative mutants of *AtVPS4*, *AtVPS24*, and *AtSNF7-1* factors in plants inhibited the activity of the isolated replicase by ∼70–75% (Fig. 2, lanes 3–8), whereas *AtVPS23-1dn* had ∼40% inhibitory effect (lanes 9–10). Expression of the full-length *AtVPS24* and *AtVPS4* did not show as much inhibitory effect on the activity of the isolated replicase as the *AtVPS24* and *AtVPS4(K178A)* dominant negative mutants (Fig. S2). Western blot analysis revealed that expression of dominant negative mutants of *AtVPS4*, *AtVPS24*, and *AtSNF7-1* factors did not affect TBSV p33 level, while inhibited the accumulation of p92^pol^ replication protein (Fig. 2, bottom panel), which could be partially responsible for the reduced activity of the TBSV replicase.

The lesser inhibitory effect of the ESCRT dominant negative mutants on the tombusvirus replicase activity (Fig. 2) than on viral RNA accumulation (Fig. 1) is likely due to the uncoupled expression of the p33 and p92^pol^ viral replication proteins and the viral replicon RNA from separate plasmids in plants used for the replicase assay, while expression of the p33/p92^pol^ is coupled to viral RNA level in the viral RNA accumulation assay.

### Deletion of *VPS23* and other ESCRT factors in yeast reduces the activity of the tombusvirus replicase *in vitro*

**Figure 3:**
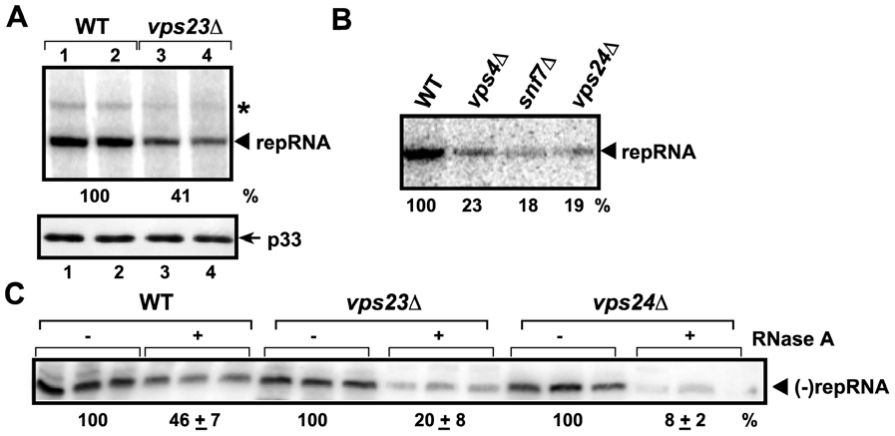
Reduced activity of the tombusvirus replicase assembled in yeast with deletion of selected ESCRT genes. (A) Denaturing PAGE analysis of *in vitro* replicase activity in the membrane-enriched fraction from wt and *vps23Δ* yeast using the co-purified repRNA. Note that this image shows the repRNAs made by the replicase *in vitro*. Asterisk marks a recombinant RNA species formed. Bottom panel shows a Western blot of p33 in the replicase preparations as seen in the top panel. (B) Decreased replication of TBSV repRNA in yeast extracts prepared from wt, *vps4Δ, snf7Δ* or *vps24Δ* yeast strains expressing p33 and p92^pol^. The yeast extracts were programmed with DI-72(+)repRNA and the radiolabeled *in vitro* repRNA products were detected via denaturing PAGE analysis. (C) Increased RNase sensitivity of TBSV minus-strand (−)repRNA during replication in a membrane-enriched replicase preparation obtained from WT, *vps23Δ* or *vps24Δ* yeast. At the end of the assays, the replicase preparations were treated with RNase A for 5 min, followed by inactivation with phenol-chloroform. The (−)repRNA protected in the replicase complex from the ribonuclease was detected using a ^32^P-UTP labeled probe. The untreated preparation was chosen as 100%. Note that the (−)repRNA is protected from RNase degradation by the membrane-associated viral replicase complex.

To further test the possible roles of ESCRT proteins in the assembly of the tombusvirus replicase complex, we used yeast model host, since yeast strains with deletion of ESCRT genes are available. To find out if the activity of the tombusvirus replicase is inhibited in *vps23Δ* yeast, we isolated the pre-assembled tombusvirus replicase from yeast cells expressing the wt p33 and p92^pol^ replication proteins and a TBSV repRNA, followed by testing for the replicase activity *in vitro*. Under the assay conditions, the pre-assembled tombusvirus replicase in the membrane-enriched fraction uses the co-purified repRNA as template for RNA synthesis, which is measured by denaturing PAGE analysis. We found that the pre-assembled tombusvirus replicase from *vps23Δ* yeast supported TBSV RNA synthesis at ∼40% of the level obtained with similar amount of replicase from the wt yeast (Fig. 3A, lanes 3–4 versus 1–2). These results have demonstrated that the tombusviral replicase was less active when formed in *vps23Δ* yeast, suggesting that Vps23p could be involved in the assembly of the viral replicase.

In the second assay, we expressed wt p33 and p92^pol^ replication proteins in yeast lacking *vps4, snf7* or *vps24*, followed by isolation of the membrane fraction carrying these viral proteins. Although the viral replication proteins associate with the membranes, they cannot form active replicase in the absence of the viral template [12],[24],[37]. Then, we added the DI-72 (+)RNA to the isolated membrane fraction to assemble the functional tombusvirus replicase *in vitro* [23], followed by replicase activity assay. In this assay, the tombusvirus replicase supports complete cycle of viral RNA synthesis *in vitro* [23]. These experiments revealed that the yeast extract prepared from *vps4Δ, snf7Δ* or *vps24Δ* yeast supported TBSV replication only at ∼20% level when compared to the wt yeast (Fig. 3B). Overall, these data demonstrated that the effect of ESCRT proteins on the tombusvirus replicase is similar in yeast and plant extracts, supporting an important role for ESCRT proteins in tombusvirus replication.

### Increased ribonuclease sensitivity of the tombusvirus replicase from *vps23Δ* or *vps24Δ* yeast

Replication of the TBSV RNA, including (−)- and (+)-strand synthesis, takes place in a membrane-bound replicase complex that provides protection against ribonucleases [23]. Since our model proposes a role of Vps23p/ESCRT proteins in facilitating the precise assembly of the tombusvirus replicase, we predicted that the tombusvirus replicase might become more sensitive to a ribonuclease if assembled in the absence of an ESCRT factor. To test this model, we isolated the membrane-bound replicase from *vps23Δ* or *vps24Δ* yeast strains expressing wt p33/p92^pol^/repRNA, which was followed by RNase A nuclease treatment that should destroy the unprotected viral RNA. Then, we used strand-specific Northern blot analysis to estimate the amount of protected (−)repRNA, which is associated with the replicase [22],[23] in the samples. These experiments revealed that only ∼20% of the (−)repRNA survived the treatment when the membrane was derived from *vps23Δ* yeast in contrast with 46% in the wt control samples (Fig. 3C). In addition, we have tested the repRNA in the membrane-fraction from *vps24Δ* yeast for ribonuclease sensitivity, since Vps24p is an important ESCRT-III factor affecting TBSV replication [5]. The protected (−)repRNA was only 8% in these samples (Fig. 3C). The simplest interpretation of the enhanced sensitivity of the (−)repRNA within the viral replicase assembled in the absence of Vps23p or Vps24p is that the viral replicase complex (possibly the whole spherule) assembles less precisely in the absence of recruitment of ESCRT proteins, thus making the viral RNA within the membrane-bound replicase-complex more accessible to a ribonuclease.

### Increased sensitivity of the minus-strands within the tombusvirus replicase to cleavage in plants expressing dominant negative mutants of ESCRT factors

**Figure 4:**
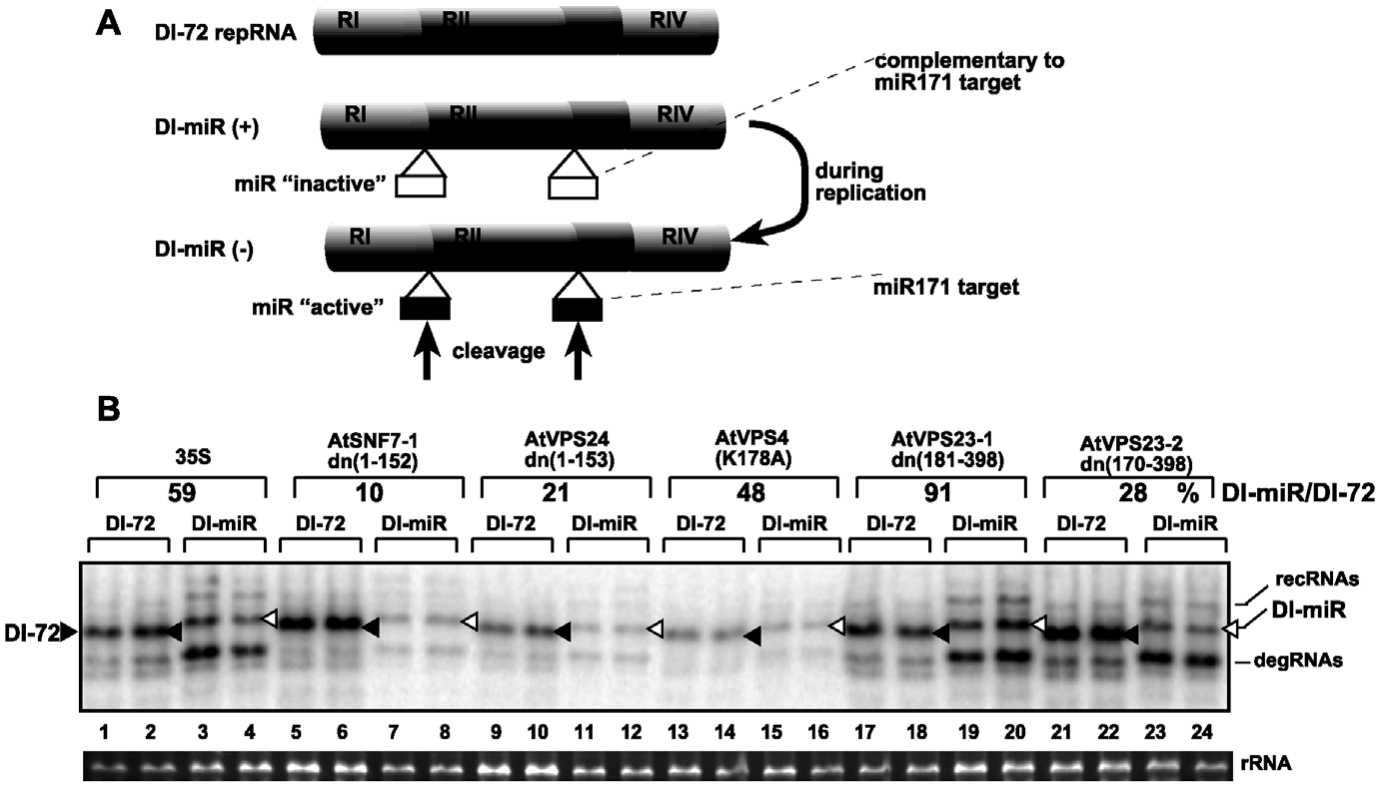
Increased sensitivity of repRNA replication to targeted degradation by RNAi in plants expressing dominant negative ESCRT-III mutants. (A) A schematic representation of constructs used as repRNAs. Two copies of the 21 nt long miR171 target sequence were inserted into DI-72 repRNA to generate DI-miR repRNA as shown. Note that both copies of the miR171 target sequence were present in the (−)strand RNA generated during repRNA replication only in plants agroinfiltrated with constructs expressing p33/p92/dominant negative ESCRT proteins and DI-72 or DI-miR repRNAs. (B) Northern blot analysis shows reduced accumulation of DI-miR repRNA in *N. benthamiana* plants expressing dominant negative ESCRT-III or Vps4p mutants. DI-72 repRNA is depicted with a black arrowhead, while DI-miR repRNA is marked with an open arrowhead. The percentage of DI-miR repRNA accumulation was calculated based on DI-72 repRNA levels (taken as 100% for each set). Note that the low level DI-miR repRNA accumulation suggests that the RNAi machinery destroyed most of the (−)repRNA present in the replicase complex. recRNA represents recombinant repRNAs, while degRNA is derived from partially degraded repRNA. Note that degRNA can replicate in plants, so it does not represent the original cleaved repRNA.

To confirm the increased sensitivity of the tombusvirus replicase complex to ribonucleases when ESCRT factors are inhibited in *N. benthamiana*, we have developed a novel approach for targeted degradation of minus-stranded RNA replication intermediate via RNA interference (RNAi). We chose the (−)repRNA as a target, since it has been shown to be always part of the membrane-bound replicase complex and it is protected from ribonucleases [22],[23]. We introduced two microRNA171 (miR171) sequences to the repRNA in such a way that the miR171 sequences were active targets to the RNAi machinery only when present in the (−)repRNA (Fig. 4).

The repRNA carrying the miR target sequences (called DI-miR, Fig. 4A) accumulated to ∼60% level of the wt repRNA (DI-72) lacking the miR171 target sequence (Fig. 4B, lanes 1-2 versus 3–4) in the control plants. On the other hand, DI-miR RNA accumulated only to 10% and 21% in plants expressing the dominant negative ESCRT-III factors, *AtVPS24*, and *AtSNF7-1,* respectively (Fig. 4B, lanes 5–12). Expression of *AtVPS4(K178A)* decreased DI-miR accumulation moderately when compared with the control plants (48% for DI-miR RNA versus 59% for DI-72 RNA, Fig. 4B).

The greatly reduced accumulation of DI-miR repRNA in comparison with DI-72 repRNA can be explained with increased sensitivity of (−)DI-miR RNA to the RNAi machinery when dominant negative ESCRT-III factors are expressed. This, in turn, supports the model that the tombusvirus replicase complexes are assembled less precisely in these plants, making them more accessible to targeted ribonucleases. Overall, these data support the role of ESCRT-III proteins in the precision/quality of viral replicase assembly.

### Binding of the p33 replication protein to Vps23p and Bro1p ESCRT proteins

**Figure 5:**
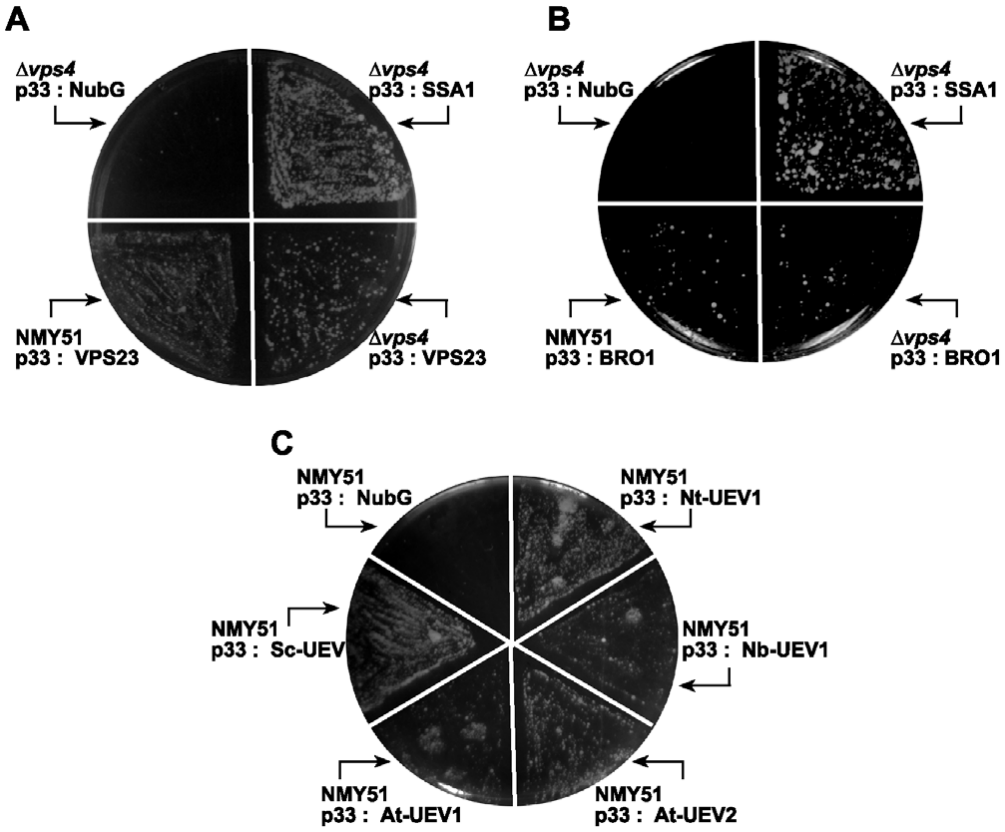
Interaction between p33 replication protein and Vps23p ESCRT-I and Bro1p accessory ESCRT proteins. The split ubiquitin assay was used to test binding between p33 and (A) Vps23p; (B) Bro1p, or (C) the N-terminal UEV domain of the yeast Sc-Vps23p or two *Arabidopsis* and two *Nicotiana* homologs in wt (NMY51) or *vps4Δ* yeast. The bait p33 was co-expressed with the shown prey proteins. *SSA1* (HSP70 chaperone), and the empty prey vector (NubG) were used as positive and negative controls, respectively.

Since the above experiments demonstrated that ESCRT proteins affect the activity of the tombusvirus replicase, we wanted to test if interaction between p33 replication co-factor and the ESCRT proteins occurs that could facilitate the recruitment of ESCRT factors for tombusvirus replication. We performed the split-ubiquitin assay, a variant of the yeast two-hybrid approach [38], which can detect protein-protein interaction on the surface of cellular membranes, where p33 is normally localized [22],[27] with selected yeast ESCRT proteins. We found that Vps23p ESCRT-I and Bro1p accessory ESCRT factors interacted with p33 in the split ubiquitin assay (Fig. 5A–B), whereas the other ESCRT proteins did not (not shown). Additionally, we found that the N-terminal UEV domain of the yeast Vps23p was sufficient for interaction with p33 (Fig. 5C). Also, the UEV domains from two *Arabidopsis* and two *Nicotiana* homologues of Vps23p interacted with the p33 replication co-factor (Fig. 5C). The interaction between p33 and either Vps23p or Bro1p was much weaker than the interaction between p33 and Ssa1p, the yeast heat shock protein 70, which is a resident protein in the tombusvirus replicase (Fig. 5A–B) [17],[37],[39]. The weak interaction with p33 suggests that Vps23p and Bro1p might only interact with p33 in a temporary fashion or only a small portion of p33 is involved in these interactions.

**Figure 6:**
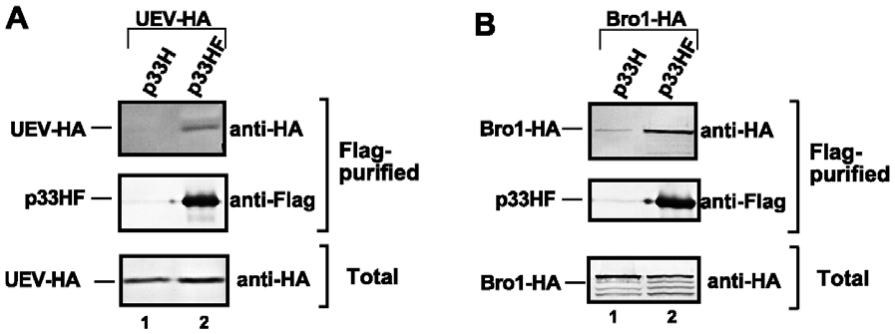
Co-purification of the p33 replication protein with the UEV domain of Vps23p ESCRT-I or Bro1p accessory ESCRT proteins. Top panels: Western blot analysis of co-purified p33 and either (A) the UEV domain or (B) Bro1p protein. The FLAG/6xHis-tagged p33HF was purified from yeast extracts using a FLAG-affinity column. UEV and Bro1p, both tagged with 6xHA, were detected with anti-HA antibody. Middle panels: Western blot of purified p33HF detected with anti-FLAG antibody. Bottom panels: Western blot of HA-tagged UEV and Bro1p in the total yeast extract using anti-HA antibody.

To further demonstrate that the interaction between p33 and Vps23p as well as Bro1p can take place in yeast cells, we co-expressed p33 replication protein tagged with FLAG and 6xHis (termed p33HF) with either the UEV domain of Vps23p or Bro1p. In this experiment, the HA-tagged UEV domain of Vps23p and Bro1p were expressed from the original chromosomal locations and the native promoters. Purification of p33HF on a FLAG-column, followed by Western analysis revealed that UEV-HA (Fig. 6A, lane 2) and Bro1-HA (Fig. 6B, lane 2) were co-purified with p33HF. Similar purification experiments on the FLAG-affinity column with p33H tagged with 6xHis only resulted in only minor amounts of nonspecifically-bound UEV-HA or Bro1-HA (Fig. 6A–B, lane 1), demonstrating that specific interaction between p33 and the UEV domain as well as Bro1p occurs in yeast.

### Redistribution of Vps23p in the presence of the p33 replication protein to the peroxisomes

**Figure 7:**
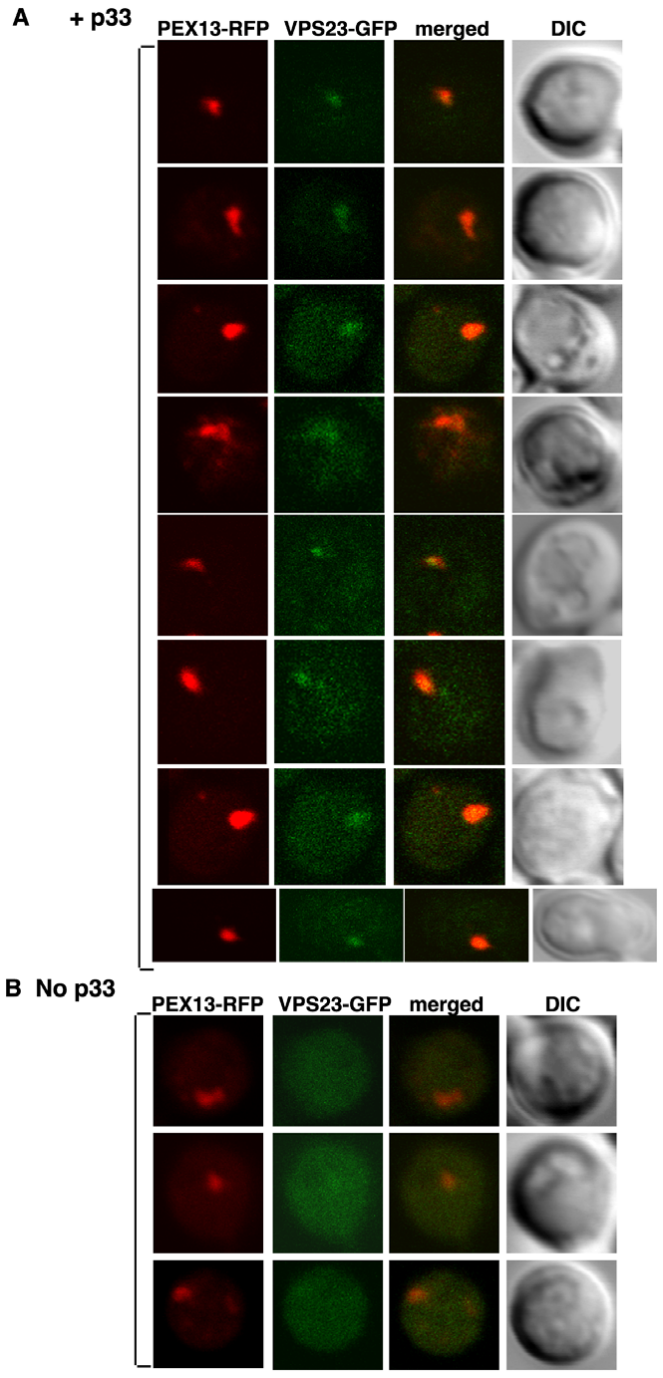
Partial re-distribution of Vps23p to the yeast peroxisomal membranes in the presence of p33. (A) Confocal laser microscopy images show the subcellular localization of Vps23p-GFP in the presence of p33 expressed from *CUP1* promoter for 15–45 minutes in yeast strain DKY79 (*VPS23:GFP, vps4Δ; vps27Δ*). The peroxisomes were visualized with Pex13p-RFP marker. The merged images show the co-localization of Vps23p-GFP and Pex13p-RFP marker. DIC (differential interference contrast) images are shown on the right. Each row represents a separate yeast cell. (B) Cytosolic localization of Vps23p-GFP in the absence of p33. Yeast was grown under similar conditions and images were taken as in panel A.

To test the subcellular compartment where p33 - Vps23p interaction takes place, we co-expressed the 6xHis-tagged p33 with Vps23p tagged with green-fluorescent protein (GFP) from its native promoter and chromosomal location in combination with Pex13p tagged with red fluorescent protein (RFP), a marker for the peroxisomal membrane [40]. Laser confocal microscopy analysis revealed that Vps23p-GFP was present in the cytosol in the absence of p33 (Fig. 7B). However, 15 min after the induction of p33 from the *CUP1* promoter, we observed the partial re-distribution of Vps23p-GFP to the peroxisomal membrane (Fig. 7A). The co-localization of Vps23p-GFP and Pex13p-RFP in cells expressing p33 is in agreement with the model that p33 is involved in re-targeting, at least temporarily, Vps23p to the peroxisomes, the sites of TBSV replication, at the beginning of replication.

## Discussion

Host factors likely play key roles during the assembly of viral replicases in infected cells [1],[2]. In this work, we have shown that a set of ESCRT proteins is critical for optimal tombusvirus replication and the assembly of the membrane-bound tombusvirus replicase complex. We found that over-expression of dominant negative mutants of ESCRT-III and Vps4p in plants reduced the accumulation of tombusvirus RNA by 10–20-fold (Fig. 1), inhibited the tombusvirus replicase *in vitro* (Fig. 2) and reduced the number of spherules formed in infected cells (Fig. S1). This inhibition seems to be specific for tombusviruses, since the distantly related TRV RNA accumulation was not inhibited in these plants. The inhibitory effect on tombusvirus replication by the over-expressed dominant negative ESCRT mutants seems to be direct, since the activity of the tombusvirus replicase was also reduced when isolated from these plants (Fig. 2). Similarly, the activity of the tombusvirus replicase assembled *in vitro* was inhibited when we used the cellular membrane fraction from yeast lacking ESCRT-III or Vps4p proteins (Fig. 3B). In addition, the replicase from *vps24Δ* yeast was more sensitive to RNase treatment than the replicase preparation obtained from wt yeast, suggesting that Vps24p ESCRT-III protein is important to assemble RNase-insensitive replicase complexes. Moreover, DI-miR repRNA carrying the miR171 target sequence in the (-)strand RNA replicated poorly in *N. benthamiana* leaves expressing dominant negative ESCRT-III mutants (Fig. 4B). These data suggest that the (-)strand repRNA in the replication intermediate within the viral replicase complex became more accessible to ribonuclease cleavage when the replicase was assembled in the presence of dominant negative ESCRT-III mutants. Altogether, the presented data support a role for ESCRT-III and Vps4p proteins in the formation of active tombusvirus replicase in plants and in yeast as well. Intriguingly, the role of ESCRT proteins seems to control the quality of the replicase complex assembly, making the viral RNAs within replicase complex more protected from ribonucleases. Based on these observations, we propose that ESCRT proteins help tombusviruses hide from host defense recognition and/or avoid the attack by the host defense during viral replication.

We also show that the recruitment of the ESCRT factors for virus replication is likely driven by interaction between the auxiliary p33 replication cofactor and Vps23p ESCRT-I protein and Bro1p accessory ESCRT protein. These interactions seem to be important for tombusvirus replication in yeast (Fig. S3) as well as in plant cells (Fig. 1). The interaction between p33 and Vps23p depends on the N-terminal UEV domain in Vps23p and p33, which is monoubiquitinated [18]. The ubiquitination of p33 may play a role in interaction with Vps23p since it has been shown that Vps23p binds to monoubiquitinated proteins [30],[41]. By binding directly to Vps23p or Bro1p, p33 might be able to recruit additional ESCRT factors for tombusvirus replication as discussed below.

Interestingly, Human immunodeficiency virus (HIV) and other enveloped retroviruses co-opt ESCRT components through direct interaction with Tsg101, the human homologue of Vps23p, and with Alix, a homologue of Bro1p [41],[42]. Tsg101 and Alix play redundant roles in this process [25]. HIV gag protein also interacts with Nedd4 E3 ubiquitin ligase protein that could complement Tsg101 or Alix during HIV budding [42]. We have shown previously that p33 can bind to Rsp5p, the yeast homologue of Nedd4 [18],[43]. This indicates that different viruses seem to exploit the ESCRT proteins through co-opting Vps23p (Tsg101), Bro1p (Alix) and/or Rsp5p (Nedd4) via direct protein-protein interactions. In addition, the ESCRT machinery is also recruited for cell division to separate the daughter cells from the mother cells through the process of cytokinesis. It has ben shown that the midbody resident protein Cep55 interacts with Tsg101 and Alix to recruit additional ESCRT proteins [41]. Interestingly, these cellular processes, such as MVB biogenesis, cytokinesis and HIV virion budding as well as the spherule formation during the assembly of the tombusvirus replicase, are based on topologically similar membrane invaginations (membrane deformation occurring away from the cytosol). Co-opting ESCRT proteins for these processes could be critical, since Snf7p and other ESCRT-III proteins have been shown to be involved in membrane deformation *in vivo* and *in vitro* as well [33],[34]. Thus, the interaction of HIV gag protein, Cep55 midbody protein and the tombusvirus p33 replication protein with members of the endosomal pathway shows close parallel and mechanistic similarities, albeit the ESCRT proteins would be used for different processes, such as either virus budding, cytokenesis or viral RNA replication.

Another novel feature is that Vps23p, in the presence of p33 replication co-factor, is shown to re-localize temporarily to the peroxisomal membrane, which represents the place of tombusvirus replicase assembly [20],[22],[27] (Fig. 7). After brief recruitment, Vps23p seems to be released from the replicase, because we did not find Vps23p in the highly-purified functional replicase complex [17] and it was not co-localized with a peroxisomal marker at latter time points after induction of p33 expression (not shown). The relatively weak interaction between Vps23p and p33 as well as the low percentage of ubiquitinated p33 [18] could be useful during virus replication to optimize the number of Vps23p recruited into each replicase complex. Indeed, based on replicases of other plus-strand RNA viruses [28], it is predicted that 100-to-200 p33 molecules are likely needed for the formation of a single replicase complex, whereas only a few Vps23p molecules should be recruited temporarily for each replicase complex. In addition, it is likely that Vsp23p and Bro1p and possibly Rsp5p could play complementary roles in recruiting additional ESCRT factors as shown in case of HIV gag for virion budding [33],[34]. Weak interactions between Vps23p - p33 and Bro1p - p33 could help recycling these host proteins that should not be present in the fully assembled replicase complex [17]. On the contrary, host factors that are permanent residents in the replicase, such as heat shock protein 70 (the yeast Ssa1p protein) [17], bind more efficiently to p33 as shown in Fig. 5.

We propose that p33 - Vps23p interaction is important for the optimal assembly of the tombusvirus replicase, because the membrane-bound tombusvirus replicase preparation obtained from *vps23Δ* yeast supported low TBSV repRNA replication (Fig. 3A). Further support on the role of p33 - Vps23p interaction in replicase assembly comes from data obtained using a membrane-enriched fraction containing the viral replicase prepared from *vps23Δ* yeast (Fig. 3C). The protection of the (-)repRNA associated with the replicase complex is likely due to the repRNA becoming inaccessible as part of the assembled replicase complex [23]. We found that the viral (−)repRNA within the replicase complex obtained from *vps23Δ* yeast was more sensitive to RNase treatment than the replicase preparation obtained from wt yeast. Thus, similar to *vps24Δ* yeast discussed above, the viral replicase, which is located inside the spherules, assembles less precisely in *vps23Δ* than in wt yeast. Overall, these data are compatible with the model that p33 - Vps23p interaction could be important during the replicase assembly process and/or affect the structure of the replicase complexes, which could determine the accessibility of the repRNA to RNases during replication. However, the effect of *VPS23* deletion (Fig. 3) or over-expression of a dominant negative mutant of Vps23 homologue in *N. benthamiana* plants (Fig. 1) was not as detrimental to virus replication as deletion of ESCRT-III or *VPS4* in yeast or over-expression of dominant negative mutants of ESCRT-III or Vps4p homologues in plants. This could be due to the redundant roles likely played by Vps23p, Bro1p and possibly Rsp5p in recruitment of ESCRT-III/Vps4p factors, based on the similar redundancy documented for subversion of ESCRT-III/Vps4p by HIV gag's interaction with Tsg101/Alix/Nedd4 [42]. Also, we do not know if the dominant negative mutants were able to block completely the function of every *VPS23* gene, since the number of *VPS23* genes is yet not known in *N. benthamiana*.

It is likely that the most important aspect of recruitment of Vps23p and Bro1p for tombusvirus replication is the possibility to co-opt additional ESCRT proteins, including the ESCRT-III factors and Vps4p. Accordingly, the *in vitro* activity of the tombusvirus replicase is 3-5-fold lower when obtained from plants expressing dominant negative ESCRT-III/Vps4p (Fig. 2) or from *vps24Δ, snf7Δ*, or *vps4Δ* yeast strains (Fig. 3B). Since the ESCRT-III factors are involved in grouping the cargo proteins together in the membrane and they have been shown to deform the membrane [33],[34], we propose that these proteins could be useful to affect the formation/structures of the spherules for virus RNA replication. Indeed, TBSV replication induces the formation of spherules (Fig. S1) [27], which are topologically related to multivesicular bodies (MVB), since both require membrane invagination into the lumen, away from the cytosol. This is opposite to the regular intracellular vesicle formation, which buds into the cytosol. Collectively, usurping a partial set of ESCRT factors by tombusvirus replication proteins might facilitate the optimal formation of active viral replicase complexes within the membranous spherules. Moreover, recruitment of Vps4p AAA ATPase [25],[35], could help re-cycling of the ESCRT factors from the replicase after the assembly. Thus, it is possible that the expression of dominant negative mutants of Vps24p and Snf7p ESCRT-III or Vps4p factors inhibit tombusvirus replication in plants by interfering with the proper assembly of the replicase complex. Accordingly, the (−)repRNA located within the viral replicase complex became more accessible to targeted ribonuclease cleavage when the replicase was assembled in the presence of dominant negative ESCRT-III mutants in plant leaves (Fig. 4B).

**Figure 8:**
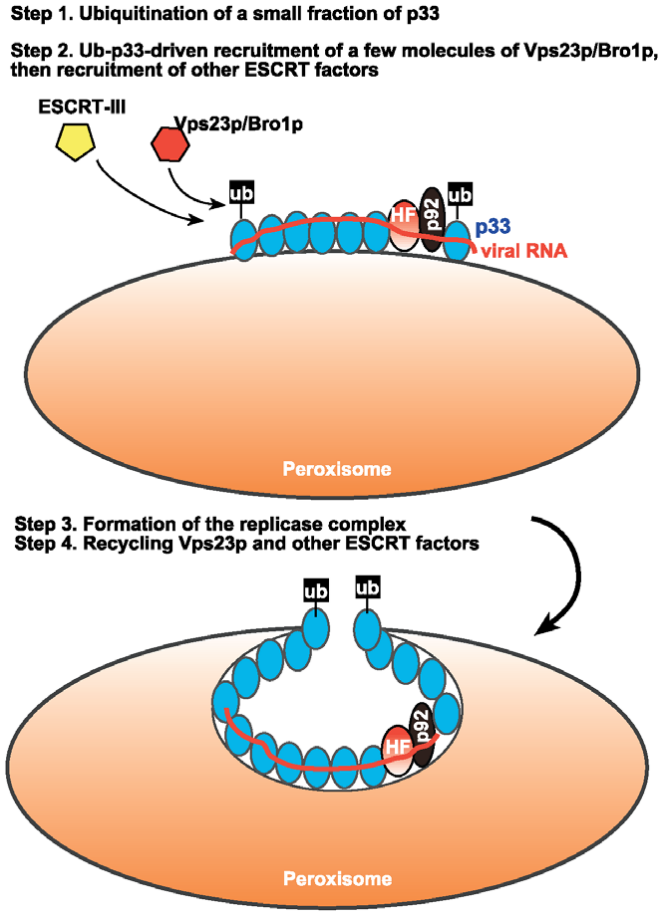
A model for the role ESCRT proteins in tombusvirus replication. Recruitment of Vps23p and/or Bro1p to the peroxisomal membrane by a small fraction of p33 is suggested to lead to the recruitment of additional ESCRT factors. Then, ESCRT-III and Vps4p factors facilitate the precise assembly of the replicase that is needed to prevent the recognition of the virus by the host surveillance system and prevent the destruction of the viral RNAs in the spherule by the host gene silencing machinery.

Based on the data presented here, we propose that the ESCRT machinery is recruited for tombusvirus replication in a unique way. The first step in recruitment of Vps23p is the ubiquitination of small percentage of p33 (Ub-p33) (Fig. 8, step 1) [18]. Then, the ubiquitinated p33 (Ub-p33) binds to the adaptor protein Vps23p (step 2) or to Bro1p accessory ESCRT protein, followed by the recruitment of ESCRT-III proteins, Snf7p and Vps24p. The ESCRT-III proteins could then help the optimal assembly of the replicase complex, facilitate the grouping of p33 molecules together in the membrane and/or promote the formation of viral spherules by deforming the membrane (membrane invagination) (step 3). Then, Doa4p deubiquitination enzyme is predicted to remove ubiquitin from Ub-p33, whereas Vps4p AAA ATPase could recycle the ESCRT proteins (step 4). Altogether, we suggest that these events could promote the optimal and precise assembly of the TBSV replicase complex, resulting in TBSV RNA replication, including (−) and (+)RNA syntheses, in a protected microenvironment and then the regulated release of the progeny (+)RNAs into the cytosol. Importantly, the precisely assembled replicase complex provides protection from recognition by the host defense surveillance system and/or viral RNA destruction by the gene silencing/RNAi machinery. Similar events might also take place in case of other (+)RNA viruses, which are also known to deform membranes and/or form spherules during replication.

## Materials and Methods

### Co-expression of CNV RNA and dominant negative mutants of selected *Arabidopsis* ESCRT proteins in *N. benthamiana*

*A. thaliana VPS4* (*At2g27600*) [44] was amplified by PCR using primers #2746 and #2749 (Table S1) and *A. thaliana* total DNA as template. This product was digested with *BamH*I and *Xho*I and ligated into similarly digested pGD [45] to generate plasmid pGD-35S-AtVPS4. To create the mutant version pGD-35S-AtVPS4(K178A), two PCR reactions were done using primer pairs #2746/#2748 and #2747/#2749 and *A. thaliana* total DNA as template. These PCR products were digested with *Nhe*I, ligated together and the product was re-amplified with primers #2746/#2749. The obtained PCR product was digested with *BamH*I and *Xho*I and cloned into pGD. The 3′ terminal portion of the two *VPS23* homologues from *A. thaliana*, namely *At3g12400* (aa 181–398, named *AtVPS23*-1dn in Fig. 1) and *At5g13860* (aa 170–368, named *VPS23*-2dn in Fig. 1) as well as the full length *At3g12400* (*AtVPS23*-1) [44], were amplified with primers #2843/#2671; #2844/#2845; and #2750/#2671, respectively. The *VPS23* PCR products were digested with *Bam*HI and *Sal*I and cloned into *Bam*HI/*Sal*I-digested pGD-L. To construct pGD-L, the leader sequence from *Tobacco etch virus* was PCR-amplified using plasmid pTEV-7DA [46] with primers #2915/#2916. The PCR product was digested with *Bgl*II and *Bam*HI and cloned into *Bam*HI-digested pGD to generate pGD-L. The ESCRT-III homologues from *A. thaliana*, namely *VPS24* [*At5g22950* (aa 1–153, named VPS24dn in Fig. 1), *At2g19830* (aa 1–152, named AtSNF7-1dn) and At4g29160 (aa 1–152, named SNF7-2dn) were amplified with primers pairs #2846/#2847; #2850/#2851; and #2852/#2853, respectively, digested with *BamH*I and *Sal*I and cloned into pGD. In addition, the 5′ terminal portion of two *VPS28* homologues (*At4g05000*, aa 1–105 and *At4g21560*, aa 1–104), and the 3′ terminal portion of a *VPS36* homologue (*At5g04920*, aa 175–440) [44] were amplified with primers #2867/#2868, #2869/#2870 and #2871/#2872, respectively and cloned into pGD using *BamHI* and *SalI*. The *BRO1* homologue from *A. thaliana* (*At1g15130*) was identified based on sequence similarity to yeast *BRO1* and *RIM20* and human *AIP1/ALIX.* A portion of *At1g15130* (aa 179–846) was amplified with primers #2883/#2884 and cloned into pGD using *BamHI* and *SalI*.

Plasmid pGD-35S-20Kstop (expressing a full length CNV RNA, but not the p20 protein) was created by PCR using primers #532/#720 and pK2/M5 20K stop [47] as template. The PCR product was digested with *Bam*HI/*Xho*I and ligated into *Bam*HI/*Xho*I-digested pGD. *A. tumefaciens* strain C58C1 transformed with pGD-35S-20Kstop or one of the pGD constructs expressing various ESCRT genes were co-infiltrated onto *N. benthamiana* leaves at OD_600_ = 0.1 and 0.5, respectively. Agroinfiltrations of *N. benthamiana* and analysis of viral RNA accumulation in the infiltrated leaves 2.5 days after infiltration were done as described [19],[48].

To launch TRV replication in *N. benthamiana*, we used the TRV plasmids pTRV1 and pTRV2 [49]. *A. tumefaciens* transformed with plasmids expressing TRV1/TRV2 and one of the dominant negative ESCRT proteins were co-infiltrated into leaves using bacterial cultures at OD_600_ = 0.1 for TRV and OD_600_ = 0.5 for the ESCRT dominant negatives as described above for CNV.

### Uncoupled expression of replicase proteins in plants and *in vitro* replicase assays

DI-72 (containing a TRSV ribozyme sequence at the 3′ end) was amplified from pYC/DI72sat [13] using primers #532/#1069 (Table S1). The PCR product was digested with *Bam*HI and *Sac*I and cloned into pGD to generate pGD/DI72sat. CNV p33 and p92 were amplified from plasmids pGBK-His33 or pGAD-His92 [12] with primers #1794/#1403 and #1794/#952, respectively. The PCR products were digested with *Bam*HI and *Xho*I and cloned into pGD-L (described above) to generate pGD-L-p33 and pGD-L-p92.

The plasmid pYC/DI72sat/2xmiR171 was designed to express a modified DI-72 repRNA containing two miR171 target sites [50], between regions I and II and regions II and III, from a 35S promoter in *N. benthamiana* plants. The orientation of both copies of miR171 sequence was to allow cleavage of the target when present in the (−)strand of the repRNA. To generate this expression plasmid, we assembled PCR products in three steps. First, we PCR-amplified region I of DI- 72 with primers #532 and #3369 from pYC/DI72sat [12],[13] followed by digestion of the PCR product with *Xba*I and gel purification of the PCR product. Second, the 3′ portion of DI-72 containing regions III, IV and the TRSV satellite ribozyme sequence [12],[13] was PCR-amplified with primers #3367 and #1069, followed by digestion with *PstI* and gel purification. Third, PCR was performed with primers #532 and #313 on pYC/DI72sat to obtain region II of DI-72, followed by digestion of the PCR product with *XbaI* and *PstI* and gel purification. The three different PCR products were ligated and used as template for a final PCR with primers #532 and #1069. The resulting PCR product was digested with *BamHI* and *SacI* and cloned into pGD plasmid resulting pYC/DI72sat/2xmiR171.

*N. benthamiana* leaves were agroinfiltrated as described [19],[48]. *A. tumefaciens* cultures containing different plasmids were combined as follows: pGD-L-p33 (OD_600_ = 0.35), pGD-L-p92 (OD_600_ = 0.15), pGD-DI72sat (OD_600_ = 0.15), pGD-p19 [to express p19 suppressor of gene silencing [48], OD_600_ = 0.15) and the above plasmids expressing the dominant negative *A. thaliana* ESCRT proteins (OD_600_ = 0.4).

Agroinfiltrated leaves were collected after 2.5 days. For analysis of repRNA accumulation, total RNA was extracted as described [5],[13]. The DI-72 repRNA was detected with a labeled RNA probe complementary to RIII/RIV(+) [5],[13]. For the analysis of the tombusvirus replicase activity, leaf samples (250 mg) were ground in liquid nitrogen and mixed with 2 ml buffer A (50 mM Tris-HCl pH 8.0, 15 mM MgCl_2_, 10 mM KCl, 2 mM EDTA, 20% glycerol, 0.3% plant protease inhibitor cocktail, 80 mM β-mercaptoethanol) [51]. The mixture was passed through a 10 ml syringe fitted with cheesecloth to trap cell debris. The clarified extract was centrifuged at 300 g for 5 min to pellet additional cell debris. The supernatant was collected and centrifuged at 21,000 g for 20 min to pellet membranes. The pellet was washed in 1 ml of buffer B+1.2 M NaCl (50 mM Tris-HCl pH 8.0, 10 mM MgCl_2_, 1 mM EDTA, 6% glycerol, 0.3% plant protease inhibitor cocktail, 80 mM β-mercaptoethanol) [51], centrifuged again and the membrane pellet was finally resuspended in 250 µl buffer B (no NaCl). 20 µl of the plant membrane fractions (containing active viral replicase) were used for *in vitro* replicase assays as described [7],[24].

### Analysis of TBSV repRNA replication in yeast and *in vitro*

Replication assays in yeast was performed as described [52]. Accumulation of DI-72 repRNA was measured by Northern blot using RNA probes complementary to region III-IV of DI-72 and to the 18S ribosomal RNA [5],[52].

*In vitro* replicase assay with membrane-enriched fraction was done as described previously [7],[24]. Note that the amount of p33 protein in each sample was adjusted to comparable levels. The *in vitro* replicase assembly assay with yeast extracts was done as described previously [23]. For the RNase protection assay, the membrane-enriched fraction from wt, *vps23Δ* and *vps24Δ* yeast strains expressing wt p33/p92^pol^/repRNA was obtained as described [12]. Then, 25 µl of the membrane-enriched preparation dissolved in buffer E [200 mM sorbitol, 50 mM Tris-HCl pH 7.5, 15 mM MgCl_2_, 10 mM KCl, 10 mM β-mercaptoethanol, 1% proteinase inhibitor mix (Sigma)] was digested with 1 µl of 20 µg/ml RNase A for 5 min. After the treatment, the RNA was extracted with phenol-chloroform, ethanol precipitated, recovered by centrifugation and analyzed in 5% polyacrylamide denaturing gel, blotted and hybridized with a ^32^P-labeled (+)DI-72 RNA probe [12].

### Protein co-purification

*S. cerevisiae* strains BRO1::6xHA-KanMX4 and UEV::6xHA-KanMX4 were generated by homologous recombination using strain BY4741 as background. PCR was performed using plasmid pYM-14 (EUROSCARF) [53] as template and primers #2493/#2494 and #2492/#2491 (Table S1), respectively. The PCR products were transformed to BY4741 and recombinant yeast colonies were selected in YPD plates supplemented with G418. Recombinant yeast strains were transformed with plasmid pGBK-33HFH or pGBK-His33 [12] and grown in minimal media supplemented with 2% glucose at 29°C. 300 µl of pelleted yeast were used to purify p33 with anti-FLAG M2 agarose as described previously [18], except that the washing steps were performed at room temperature. P33 was detected with anti-FLAG antibody (1/5,000 dilution) and AP-conjugated anti-mouse antibody (1/5,000). Bro1-6xHA and UEV-6xHA proteins were detected with anti-HA antibody from rabbit (Bethyl; 1/10,000 dilution) and AP-conjugated anti-rabbit (1/10,000) followed by NBT-BCIP detection.

### Analysis of protein interactions

The split-ubiquitin assay was based on the Dualmembrane kit 3 (Dualsystems biotech). pGAD-BT2-N-His33 has been described previously [18]. pPR-N-VPS23 and pPR-N-ScUEV were generated by PCR using yeast genomic DNA and primer pairs of #2252/#2046 and #2252/#2292 (Table S1), respectively. The PCR products were digested with *EcoR*I and *Nhe*I and cloned into pPR-N-RE [18]. pPR-C-BRO1 was obtained by PCR using yeast genomic DNA and primers #2053/#2054. The product was digested with *Bam*HI and *Nhe*I and cloned into pPR-C-RE [18].

*A. thaliana* UEV-1 (At3g12400; aa1-186) and UEV-2 (At5g13860; aa1-175) were amplified from genomic DNA with primers #2669/#2670 and #2984/#2985 respectively, digested with *Bam*HI and *Sal*I and cloned into pPR-N-RE digested with *Bam*HI/*Sal*I or *Bgl*II/*Sal*I. *Nicotiana* sp homologues of Vps23-UEV were amplified from *N. benthamiana* or *N. tabacum* genomic DNA with primers #2986 and #2987 (based on accession # EB680173; nt 208-750), digested with *Bgl*II and *Sal*I and cloned into pPR-N-RE digested with *Bgl*II/*Sal*I.

Yeast strain NMY51/vps4Δ::URA3 was created by homologous recombination using the URA3 gene, which was amplified from plasmid pCM189 [54] with primers #2446 and #2447. The PCR product was transformed into yeast strain NMY51 (Dualsystems) and the recombinants selected on Ura- plates [18]. NMY51 or NMY51/vps4Δ::URA3 were transformed with pGAD-BT2-N-His33 and pPR constructs. Transformed colonies were selected in Trp-/Leu- plates. Yeast colonies were re-suspended in a small volume of water and streaked onto Trp-/Leu-/His-/Ade- plates to score interactions.

### Confocal laser microscopy

*S. cerevisiae* strain DKY79 (VPS23-GFP, *vps27Δ, vps4Δ*), expressing the GFP tagged Vps23p from the native promoter and chromosomal location [55] was transformed with pYC-CUP-Flag33 and/or pGAD-pex13-RFP. To create pYC-CUP-Flag33, Flag-tagged p33 was amplified from pGBK-33HFH with primers #2450/#992B (Table S1), digested with *NcoI*/*PstI* and cloned into similarly digested pGBK-His33/CUP1 [56]. The resulting plasmid was used as template for a PCR with primers #2753/#1403. The product was digested with *Nhe*I/*Xho*I and cloned into *Spe*I/*Xho*I digested pYC2/CT (Invitrogen) generating pYC-CUP-Flag33. *PEX13* ORF was amplified by PCR using primers #1277/#1278 and pGAD-pex13-CFP [22] as template. *RFP* ORF was amplified from genomic DNA of a pex3-RFP yeast strain [40] using primers #2691/#2663 (Table S1). Both PCR products were digested with *Bgl*II, ligated and reamplified with primers #1277 and #2663. This product was digested with *Hind*III and *Bam*HI and cloned into similarly digested pGAD H- [12] to generate pGAD-pex13-RFP. The transformed yeast strains were grown at 29°C in minimal media supplemented with 2% glucose. Yeast cells were imaged with Olympus FV1000 confocal laser scanning microscope [20] within 15–45 minutes after addition of 50 mM CuSO_4_ to induce p33 expression.

## Supporting Information

Figure S1 Reduced number of tombusvirus-induced spherules in plant cells expressing dominant negative mutants of Snf7-1p and Vps4p. Representative electron microscopic images of portions of *N. benthamiana* cells. Several characteristic virus-induced spherules are marked with arrowheads. These spherules are formed via membrane invagination into peroxisomal or ER-derived membranes. Panels I and II show control samples, which were obtained from leaves either infected with CNV gRNA or agroinfiltrated to express p33/p92/DI-72 repRNA. Panel III shows a magnified portion of panel II to visualize ∼20 individual spherules within the membranous structure. Note that, in addition to the reduced numbers (not shown), the sizes of the spherules are very variable in cells over-expressing the dominant negative Snf7-1p (panel IV) when compared with the control infections (panels I and II). The expression of the dominant negative Vps4p mutant made portions of the cells containing irregular membranes and we could not definitively identify virus-induced spherules (panel VI).
(2.72 MB TIF)

Figure S2 The in vitro activity of the isolated tombusvirus replicase preparations from *N. benthamiana* plants expressing full-length ESCRT factors. Denaturing PAGE of in vitro replicase activity in the membrane-enriched fraction from co-infiltrated leaves expressing p33, p92^pol^, DI-72 repRNA, p19 (suppressor of gene silencing) and the shown ESCRT factors using the co-purified repRNA template.
(0.05 MB PDF)

Figure S3 The effect of *bro1Δ* and *vps23Δ* on tombusvirus RNA accumulation in yeast. Total RNA was extracted from yeast 24 hours after inducing repRNA replication. The accumulation of (+)repRNAs was measured by Northern blotting, whereas the ribosomal RNA (rRNA) was used as a loading control (not shown). Each experiment was done at least six times. Overall the result indicates that the lack of *BRO1* and *VPS23* ESCRT genes inhibits repRNA accumulation, suggesting that these genes play a role in tombusvirus replication.
(0.01 MB PDF)

Protocol S1 Supplementary materials and methods.
(0.08 MB PDF)

Table S1 List of primers used in this study.
(0.04 MB PDF)
