# Supplementary figures and images for: A Unique Role for the Host ESCRT Proteins in Replication of Tomato bushy stunt virus
Source: PLoS Pathog. 2009 Dec 24;5(12):e1000705. doi: 10.1371/journal.ppat.1000705 (PMC2791863; doi:10.1371/journal.ppat.1000705)

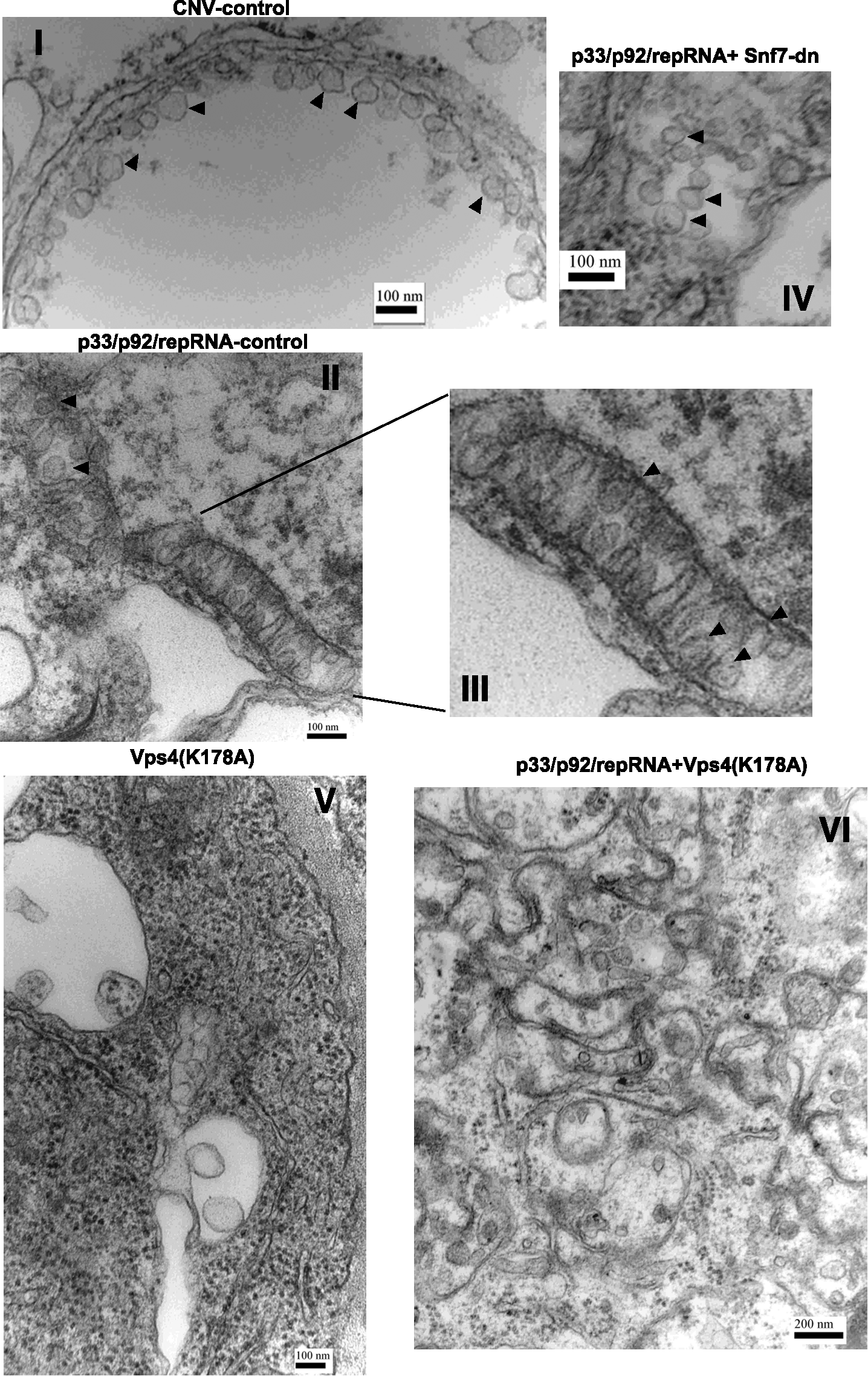

Supplement: Figure S1 — Reduced number of tombusvirus-induced spherules in plant cells expressing dominant negative mutants of Snf7-1p and Vps4p. Representative electron microscopic images of portions of N. benthamiana cells. Several characteristic virus-induced spherules are marked with arrowheads. These spherules are formed via membrane invagination into peroxisomal or ER-derived membranes. Panels I and II show control samples, which were obtained from leaves either infected with CNV gRNA or agroinfiltrated to express p33/p92/DI-72 repRNA. Panel III shows a magnified portion of panel II to visualize ∼20 individual spherules within the membranous structure. Note that, in addition to the reduced numbers (not shown), the sizes of the spherules are very variable in cells over-expressing the dominant negative Snf7-1p (panel IV) when compared with the control infections (panels I and II). The expression of the dominant negative Vps4p mutant made portions of the cells containing irregular membranes and we could not definitively identify virus-induced spherules (panel VI). (2.72 MB TIF) [file ppat.1000705.s001.tif]

Replicase assay

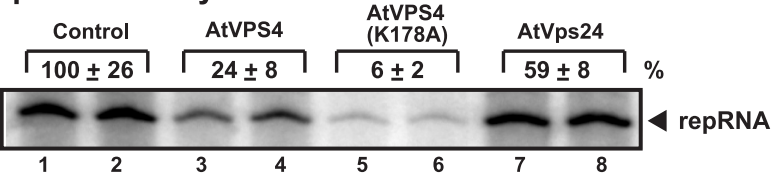

Supplement: Figure S2 — The in vitro activity of the isolated tombusvirus replicase preparations from N. benthamiana plants expressing full-length ESCRT factors. Denaturing PAGE of in vitro replicase activity in the membrane-enriched fraction from co-infiltrated leaves expressing p33, p92pol, DI-72 repRNA, p19 (suppressor of gene silencing) and the shown ESCRT factors using the co-purified repRNA template. (0.05 MB PDF) [file ppat.1000705.s002.pdf]

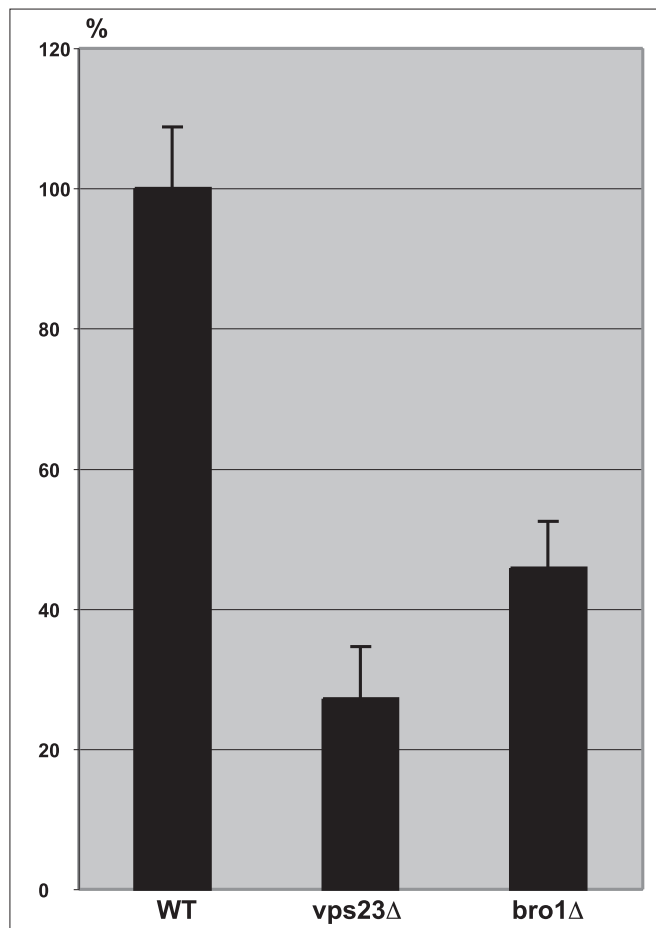

Supplement: Figure S3 — The effect of bro1Δ and vps23Δ on tombusvirus RNA accumulation in yeast. Total RNA was extracted from yeast 24 hours after inducing repRNA replication. The accumulation of (+)repRNAs was measured by Northern blotting, whereas the ribosomal RNA (rRNA) was used as a loading control (not shown). Each experiment was done at least six times. Overall the result indicates that the lack of BRO1 and VPS23 ESCRT genes inhibits repRNA accumulation, suggesting that these genes play a role in tombusvirus replication. (0.01 MB PDF) [file ppat.1000705.s003.pdf]
