# Supplementary material for: A Unique Role for the Host ESCRT Proteins in Replication of Tomato bushy stunt virus
Source: PLoS Pathog. 2009 Dec 24;5(12):e1000705. doi: 10.1371/journal.ppat.1000705 (PMC2791863; doi:10.1371/journal.ppat.1000705)
Supplement: Protocol S1 — Supplementary materials and methods. (0.08 MB PDF) [file ppat.1000705.s004.pdf]

SUPPLEMENT:

Materials and Methods. Electron microscopy. *N. benthamiana* leaves were agroinfiltrated with a construct expressing CNV 20k stop or a combination of *A. tumefaciens* cultures expressing p33, p92, p19 proteins, DI-72 RNA and the ESCRT mutants. Leaf samples were fixed 2.5 days after agroinfiltration with a fixing buffer containing 0.1 M KH_2PO_4, pH 6.8, 3.5% glutaraldehyde and 1% paraformaldehyde. The leaves were injected with the fixing buffer using a syringe (without needle) and subsequently cut and sectioned into 1x5 mm strips. The leaf sections were immersed in the fixing buffer and incubated overnight at 4º C. Leaf sections were washed three times for 10 minutes in 0.1 M KH_2PO_4 pH 6.8, plus 5% glucose, then treated with 1% OsO_4 for 2 hours at room temperature. Sections were washed in distilled water for 5 min and dehydrated sequentially using 50%, 70%, 80% and 90% ethanol washes for 10 min each at room temperature, followed by two 100% ethanol washes for 20 min and two propylene oxide (PO) washes for 15 min. Samples were gradually infiltrated in 50/50 epon-araldite resin/PO over night, 75/25 resin/PO for 4 hours and then 100% resin for 4 hours under vacuum. Samples were finally embedded in resin and incubated for 48 hours at 60º C for resin polymerization. After sectioning and mounting in copper grids, samples were stained with uranyl-acetate and lead-citrate and imaged in a Philips Biotwin 12 Transmision electron microscope. The images were cropped using Photoshop software.
