# Supplementary material for: A Unique Role for the Host ESCRT Proteins in Replication of Tomato bushy stunt virus
Source: PLoS Pathog. 2009 Dec 24;5(12):e1000705. doi: 10.1371/journal.ppat.1000705 (PMC2791863; doi:10.1371/journal.ppat.1000705)
Supplement: Table S1 — List of primers used in this study. (0.04 MB PDF) [file ppat.1000705.s005.pdf]

Table S1. List of primers used in this study

0532	GGCGGGATCCGGAAATTCTCCAGGATTTCTC
0313	CCCAACAAGAGTAACCTGTATGCTATGCCA
0720	CCCGCTCGAGGGGCTGCATTTCTGCAATGTTC
0952	CCCGCTCGAGTCATGCTACGGCGGAGTCAAGGA
0992B	GAGCTGCAGCTATTTCACACCAAGGGA
1069	CCGGTCGAGCTCTACCAGGTAATATACCACAACGTGTGT
1277	CGGCAAGCTTACCATGTCATCCACAGCAGTACCACGA
1278	CGGGCTCGAGGTGTGTACGCGTTTCATCATCAACA
1403	GCCGCTCGAGCTATTTCACACCAAGGGACTCA
1794	GCGGGATCCATGGATACCATCAAGAGGATG
2046	CGACCTCGAGTCAGCTAGCCGATAACGGTGAGGTGATTCG
2053	GCTCCTCGAGGGATCCATGAAACCTTACTTATTTGACCTAA
2054	CGACCTCGAGCTAGCTAGCACTGCTGTATTTGGAGTACATATT
2252	GCCGAATTCATGTCTGCAAACGGCAAGATCTCTG
2292	GCTCCTCGAGTTAGCTAGCCAATGGCGGTTTTAGGTGTGG
2446	AATAAGAACTTCATGATATCGAACGATTTAATCGTGGGTTGAGGCCCTTTCGTCTTCAAG
2447	TTTCATGTACACAAGAAATCTACATTAGCACGTTAATCAATTGAAGCTCTAATTTGTGAG
2450	GGCAAGCTTACCATGGGTCGGGATTACAAGGAC
2491	TATTTTTTATGGCACTTCGGCGATGCGAAAGAAAGTGAGTCAATCGATGAATTCGAGCTC
2492	ACTGCCCCCAAAGCCCAAGTCTCCACACCTAAAACCGCCATTGCGTACGCTGCAGGTCGA
2493	ACCCTCTGTTTTGATGAAAATATGTACTCCAAATACAGCAGTCGTACGCTGCAGGTCGA
2494	AAAAAAAGCTACAATAAAATTAAGAAATAAGAAATGCACCTAATCGATGAATTCGAGCTC
2663	GCGGATCCGCTAGCGGCGCCGGTGGAGTGGCG
2669	GCGGATCCGAATTCCCGATGGTTCCCCCGCCGTC
2670	GCCGAGCTCGTCGACTCAGAACGGTCTAGGCAATGAC
2671	GCCGAGCTCGTCGACTCATGAATGTAACCTACCTGC
2691	CGGAGATCTATGGCCTCCTCCGAGGAC
2746	GCGAATTCGGATCCATGGGTCATCATCATCATCATCATTACAGCAATTTCAAGGAACAAG
2747	GCGGATCCGCTAGCTATTTGGCTAAGGCTGTTGC
2748	CGGCTCGAGGCTAGCTCCTGTTCCAGGAGGCCCA
2749	CGGCTCGAGGAGCTCTCAACCTTCTTCTCCAAACTC
2750	GGCGAATTCGGATCCACCATGGGTCATCATCATCATCATCATGTTCCCCCGCCGTCTA
2753	GCCGCTAGCGACATTTGGGCGCTATACGT
2843	GGCGAATTCGGATCCATGGGTCATCATCATCATCATCATCGACCTCCTTCGGCTGA
2844	GGCGAATTCGGATCCATGGGTCATCATCATCATCATCATCCTTCGATCGGATCTGGA
2845	GCCGAGCTCGTCGACTCACGAATGCAACCTAGCTG
2846	CGCGGATCCATGGGTCATCATCATCATCATCATATGGAGAGAGTGATGAACATC
2847	GCCGTCGACTCAAGCATTGTCAATGGCCTCATTC
2850	CGCGGATCCATGGGTCATCATCATCATCATCATATGTTTATGAATCGGCTATTCG
2851	GCCGAGCTCGTCGACTCAATCATTAGCCCCAAATGGAG
2852	CGCGAATTCGGATCCATGGGTCATCATCATCATCATCATATGATGAATCGGCTATTCGG
2853	GCCGAGCTCGTCGACTCAGTCAGCCGCTGCCCCCA
2867	GCGAATTCGGATCCATGGGTCATCATCATCATCATCATATGGAGGTCAAATTATGGAAC
2868	GCCGAGCTCGTCGACTCATGCAACGGTAGCTCGGTG
2869	GCGAATTCGGATCCATGGGTCATCATCATCATCATCATGAGGTCAAGTTATGGAATG
2870	GCCGAGCTCGTCGACTCACGAAGCAGCAGCTCTATG
2871	GCGAATTCGGATCCATGGGTCATCATCATCATCATCATGGATTGGCTGGGATTTTGAG
2872	GCCGAGCTCGTCGACTCATACAGACAACAAACTGGTCG
2883	CGCGGATCCATGGGTCATCATCATCATCATCATACCGTGGATGTGTCGGTTG
2884	GCCGTCGACTCATTGCCTGTAGTATCCTCCAC
2915	GCCGAGATCTTCTCAACACAACATATACAAAAC
2916	GCCGGATCCGGCTATCGTTCGTAAATGGTG
2984	GCCGAGCTCGTCGACTCACCAGATCCGATCGAAGGAG
2985	GGCGGATCCATGGGTCATCATCATCATCATCATAGATCTGCTCCTCCTCCGGCGAAG
2986	GGCGGATCCATGGGTCATCATCATCATCATCATAGATCTATGCCGCCACCGAATTCC
2987	GCCGAGCTCGTCGACTCAACTCGTACTGGGCGGAGG
3367	CGGCTGCAGCTATAACCGCGCCGAGTTAGTAGCGAGTAAGACAGACTC
3369	GCGGTCTAGAACTAACTCGGCGCGGTTATAGCATGTCGCTTGTTTGTTGG
